# PD-1 blockage combined with vaccine therapy can facilitate immune infiltration in tumor microenvironment of Lynch syndrome colon cancer

**DOI:** 10.3389/fgene.2022.877833

**Published:** 2022-09-08

**Authors:** Kai Ye, Wenjin Zhong, Pengcheng Wang, Yanxin Chen, Pan Chi

**Affiliations:** ^1^ Department of Gastrointestinal Surgery, Second Affiliated Hospital of Fujian Medical University, Quanzhou, Fujian Province, China; ^2^ Department of Colorectal Surgery, Union Hospital Affiliated of Fujian Medical University, Fuzhou, Fujian Province, China

**Keywords:** Lynch syndrome, immune checkpoint-inhibitors, colon cancer, tumor vaccine, tumor microenvironment

## Abstract

**Background:** Lynch syndrome is a genetic disease resulting from mismatch repair gene mutation. Vaccine therapy can enhance the immunogenicity of Lynch syndrome and improve the therapeutic efficacy of immunotherapy. However, there is no approved Lynch syndrome vaccine coming onto the market.

**Methods:** Herein, we used gene knockdown method to construct Lynch syndrome cell model, paving way for us to develop Lynch syndrome tumor lysate vaccine. Then the isograft technique was employed for constructing the tumor-bearing mouse model of Lynch syndrome. And this isograft model was treated with PD-1 monoclonal antibody and tumor vaccine, respectively. Flow cytometry was used for detecting the proportion of immune cells and immunosuppressive cells, and ELISA was used for detecting the contents of chemokines and cytokines in the blood circulation system and tumor tissues of mice. Finally, IHC was used to detect the effects of tumor vaccines as well as PD-1 antibody on tumor tissue proliferation and angiogenesis.

**Results:** The results demonstrated that tumor vaccine could prolong the overall survival of mice, and improve the disease-free survival rate of mice. The vaccine could increase the proportion of inflammatory cells and decrease the proportion of anti-inflammatory cells in the blood circulation system of mice. In addition, tumor vaccine could also improve inflammatory infiltration in the tumor microenvironment and reduce the proportion of immunosuppressive cells. The results of IHC showed that tumor vaccine could inhibit angiogenesis and tumor cell proliferation in mouse tumor tissues.

**Conclusion:** In colon cancer associated with Lynch syndrome, tumor vaccine can hinder the growth of tumor cells, and assist immunotherapy whose therapeutic effect on this kind of cancer is thus enhanced.

## Introduction

Immune checkpoint-inhibitors (ICIs) have been the most common clinical treatment for solid tumors, which are extensively applied in treating many cancers including melanoma, breast cancer and lung cancer due to the good therapeutic effect (Vaddepally et al., 2020). At present, many ICIs have got market approval, such as ipilimumab, nivolumab and pembrolizumab, which have shown great efficacy in clinical practice. For example, nivolumab combined with chemotherapy can prolong the median survival time of patients with esophageal cancer from 11.1 months (chemotherapy) to 13.1 months (nivolumab plus chemotherapy) (Janjigian et al., 2021). In a clinical study of patients with advanced triple-negative breast cancer, the researchers found that for patients with high tumor mutational burden (TMB) whose TMB over 10, pembrolizumab increased the objective response rate to 14.3% compared with chemotherapy (8.3%) (Winer et al., 2020). There are many ways by which ICIs work. Currently, ICIs drugs mainly target common immune checkpoints such as CTL4, PD-1 and PD-L1. By binding to the above targets, ICIs are able to mask immunosuppressive signals, thereby inhibiting the immune escape of tumor cells, alleviating the state of T cell depletion in the tumor microenvironment (TME) and improving the activity of the immune system (Jia et al., 2020). Much as ICIs have shown promising therapeutic effects in clinical practice, many patients do not respond well to the treatment with ICIs as a result of T cell depletion and poor immunogenicity of cancer cells (Blank and Mackensen, 2007). The way to improve the therapeutic effect of ICIs in such kind of patients is a pressing matter for scientists and clinicians.

Lynch syndrome is an autosomal dominant hereditary disease resulted from mutations in mismatch repair genes, and patients with Lynch syndrome have a higher risk of being diagnosed with colorectal and endometrial cancer. Lynch syndrome is the most common genetic syndrome giving rise to colorectal cancer, accounting for 3% of newly diagnosed colorectal cancer incidences (Sinicrope, 2018). Because patients with Lynch syndrome have inherited mutations in mismatch repair genes, they often exhibit microsatellite instability (MSI) and TMB-H after cancer onset, and interestingly, these two phenotypes are considered to have a fair response to immunotherapy (Rizzo et al., 2021). Current clinical practice has found favorable efficacy of immunotherapy in patients with Lynch syndrome. The results of ICIs treatment in patients with pan-cancer Lynch syndrome showed that the objective response rate is 94% (16/17), of which 94% (15/16) have persistent response without disease progression and relapse (Bari et al., 2020). Since cancer patients with Lynch syndrome have a good drug response to ICIs, the way to improve the therapeutic effect of ICIs on those patients is an urgent clinical problem to overcome, which is also the key to enhance the survival rate of patients with Lynch syndrome.

The tumor vaccine is a vaccine designed by using tumor tissue antigens, which contains tumor-specific antigens, aiming at eliciting an immune response against tumor antigens (Buonaguro and Tagliamonte, 2020). Recently, researchers have found that the therapeutic effect of ICIs can be enhanced once the patient’s immune system is mobilized after the inoculation of tumor vaccine. For example, TAS0314 long-chain peptide vaccine has been found to have a synergistic anti-tumor immune effect with PD-1/PD-L1, which can enhance the therapeutic effect of PD-1/PD-L1 blockage by promoting the infiltration of cancer-specific cytotoxic T lymphocytes (CTLs) in tumor tissues (Tanaka et al., 2020). Since tumor cells from patients with Lynch syndrome are immunogenic and have a large number of mutation sequences, we speculated that tumor vaccines based on Lynch syndrome cells may help stimulate the patient’s immune system and promote anti-tumor immunity to improve the sensitivity of ICIs. In this study, we first used gene knockout method to construct mouse MC38^Mlh1KD^ cells, which underwent homotransplantation to construct a mouse model of Lynch syndrome. Then mice were treated with tumor vaccine, and PD-1 blockage separately to explore the synergy of tumor vaccine on PD-1 blockage treatment. This study investigated the synergy of tumor vaccine on PD-1 blockage therapy for colon cancer in Lynch syndrome in the hope of providing more theoretical basis and reference for treating Lynch syndrome clinically.

## Materials and methods

### Cell culture and vaccine preparation

The murine colon carcinoma cell line MC38 (BNCC337716), purchased from BeNa Culture Collection (BNCC), was cultured in DMEM-H medium containing 10% FBS along with 1% P/S. The cells were cultured in a constant temperature incubator at 37°C with 5% CO_2_. The lentiviral vector encoding Mlh1 shRNA was synthesized by GenePhama (China) and transfected into MC38 cells to construct dMMR CRC model cells with stable knockdown of Mlh1 (MC38^M1h1 KD^).

The tumor vaccine was prepared based on well-immunoreactive dMMR-type mouse tumor cells (MC38^Mlh1 KD^). MC38^Mlh1 KD^ cells were collected, and repeatedly frozen and thawed between −80°C and 37°C (5 minutes each, 4 cycles), followed by one heat shock treatment (42°C, 5 min). Lysates from collected tumor cells were stored at −80°C.

### *In Vivo* animal experiment

To detect the specific effect of tumor vaccine treatment on the survival time and tumor tissues of mice, 30 C57/BL6 mice aged 6–8 weeks acquired from Nanjing Institute of Model Animals were equally divided into two groups. The control group were injected with 2 × 10^6^ (Sinicrope, 2018) MC38^Mlh1 wildtype^ cells in the right lower limb of mice, while the experimental group were injected with 2 × 10^6^ (Sinicrope, 2018) MC38^Mlh1KD^ cells. These mice were all treated with vaccine (injected with tumor cell lysate, 10 mg/kg bw, s.c., biweekly). The survival time experiment was enrolled 20 C57/BL6 mice with 10 mice in each group. Treatment continued until 70 days or until the mice died. The tumor tissues detection experiment was enrolled 10 C57/BL6 mice with 5 mice in each group. Mice were euthanized after 35 days of treatment, and then measured the tumor tissues volume and weight.

To detect the effect of tumor vaccine and PD-1 treatment on the survival time of mice, 40 C57/BL6 mice aged 6–8 weeks acquired from Nanjing Institute of Model Animals were injected with 2 × 10^6^ (Sinicrope, 2018) MC38^Mlh1KD^ cells in the right lower limb of mice to construct an isograft dMMR colon cancer model. The mice were divided into control group (treated with IgG) (Rockland Immunochemicals Inc., Boyertown, PA, USA), vaccine treatment group (injected with tumor cell lysate, 10 mg/kg bw, s.c., biweekly), PD-1 treatment group (injected with murine PD-1 monoclonal antibody 2.5 mg/kg bw, i.v., biweekly) or double treatment group (simultaneously injected with 10 mg/kg bw tumor cell lysate and 2.5 mg/kg PD-1 monoclonal antibody) according to different treatment methods, with 10 mice in each group. Treatment continued until 70 days or until the mice died.

To detect the changes of TME in mouse tumor tissues, 20 C57/BL6 mice aged 6–8 weeks obtained from Nanjing Institute of Model Animals were divided into groups according to the above treatments, 5 mice in each group. Mice were euthanized after 35 days of treatment, and whole blood of mice was obtained by eyeball blood sampling method. The tumor tissues were used for subsequent detection.

Tumor length and width were measured to calculate tumor volume by using the mathematical formula: volume (mm^3^) = (width^2^ (Janjigian et al., 2021) × length)/2.

### Flow cytometry

Blood samples were obtained from previously collected whole blood**.** Blood samples were collected 150 μl/time from each mouse once every 2 weeks by eyeball blood sampling method. For flow cytometry detection of tumor tissues, tumor tissues were first collected from mice, mashed and passed through a 100-μm cell strainer and prepared into single cells suspensions. A panel of conjugated antibodies (Abcam, UK) was subsequently used for staining: FITC Anti-CD3 antibody, APC Anti-CD8 alpha antibody, PE Anti-CD4 antibody, PE Anti-NKR-P1C (NK1.1), FITC Anti-CD11b antibody, APC Anti-Ly6g antibody (gr1), FITC Anti-CD19 antibody, FITC Anti-CD83 antibody, PE/Cy7^®^ Anti-PD1 antibody, PerCP/Cy5.5^®^ Anti-PD-L1 antibody, PE Anti-LAG-3 antibody, PE Anti-CTLA4 antibody. Negative controls were stained by lymphocytes of the appropriate isotype. Flow cytometry was performed using BD FACSVerse™ and analysis of flow cytometric data was performed using BD FACSuite software.

### ELISA test

Under the instructions of manufacturer, ELISA kits (MultiSciencesBiotech, China): Mouse TNF-a ELISA Kit, Mouse CCL4/MIP-1β ELISA Kit, Mouse IL-10 ELISA Kit, Mouse IL-13 ELISA Kit, Mouse CCL11/Eotaxin ELISA Kit, Mouse CCL5/RANTES ELISA Kit were employed for measuring expression of immune factors in serum.

### Immunohistochemistry test and hematoxylin-eosin staining

Fresh tumor tissues were taken and fixed by immersion in formalin solution, and the formalin-fixed tissues were subsequently sectioned using paraffin embedding. Hematoxylin-eosin (H&E) staining was used for histopathological observation. For IHC staining, antigen recovery was first performed in EDTA buffer at pH = 9.0, followed by blocking of endogenous peroxidase activity as well as non-specific binding using ltraSen-sitive S-P kits (Maixin Biotechnology, China). Sections were incubated with rabbit anti-human anti-PD-L1 (Abcam, United Kingdom), rabbit anti-human anti-Ki-67 (Abcam, United Kingdom), and rabbit anti-human anti-CD31 monoclonal antibody at 4°C. Secondary antibody-horseradish peroxidase co-incubation was subsequently used (Maixin Biotechnology, China), and 3,3-diaminobenzidine-tetrahydrochloric acid was used as chromogen for coloration (Maixin Biotechnology, China).

The scoring criteria for staining intensity were as follows: staining (0), weak staining (1), moderate staining (2) and strong staining (3). The scoring criteria for the positive proportion of stained tumor cells were as follows: 0 (0%), 1 (1%–10%), 2 (11%–50%), 3 (51%–80%), and 4 (>80%). The staining results were evaluated semiquantitatively by multiplying the staining intensity by the percentage of positive tumor cells. Then the sections were scored by two experienced pathologists, and the average of the scores was selected as the final result. For evaluation of microtubule density, the number of microvessels per unit area was calculated using 3 random fields after staining with CD31, and the mean value was seen as the final result.

### Quantitative real-time polymerase chain reaction

Trizol reagent (Takara, Japan) was employed for the extraction as well as purification of total DNA. qRT-PCR was performed in triplicate in the ABI 7500 fast real-time PCR System (Applied Biosystems, United States). The relative expression level of Mlh1 was calculated through normalization to GAPDH internal controls. The following primers were used for PCR detection:

Mlh1: forward: 5′-CTC​CAA​GAT​GAG​GCT​GTA​GGA​A-3′; reverse: 5′-CCT​ATG​AGA​TGG​AAG​GCA​AGA-3′; GAPDH forward: 5′-CTG​GGC​ACT​GAG​CAC​C-3′; reverse: 5′-AAG​TGG​TCG​TTG​AGG​GCA​ATG-3′.

### Western blot

The extraction of total protein and the measurement of protein concentration were performed respectively by using radioimmunoprecipitation assay lysis buffer (Beyotime, China) and the BCA protein assay kit (Thermo Fisher Scientific, United States), respectively. Protein samples were separated by 10% SDS-PAGE and then transferred onto Polyvinylidene fluoride membrane (Millipore, United States). Afterwards, the membrane was blocked in 5% skim milk at 37°C for 1 h and then incubated with the primary antibodies at 4°C overnight. After incubating with Horseradish Peroxidase-conjugated secondary antibody for 1 h at 37°C, the membrane was washed in TBST and prepared for signal detection. The bands were visualized using an ECL chemiluminescent detection system (Thermo Fisher Scientific, United States). The primary antibodies and second antibody were purchasing from Abcam (United Kingdom), as anti-Mlh1 antibody (ab92312), anti-GAPDH antibody (ab9485), goat anti-rabbit IgG H&L antibody (ab6721).

### Data analysis

In this paper, Graphpad Prism 8 was used to plot the data and data analysis, and one-way ANOVA was used for significance test in advance, and Student’s *t*-test was used for intergroup analysis. *p* < 0.05 was considered statistically significant.

## Results

### Tumor vaccines combined with ICIs can remarkably prolong the survival time of Lynch syndrome model mice

**FIGURE 1:**
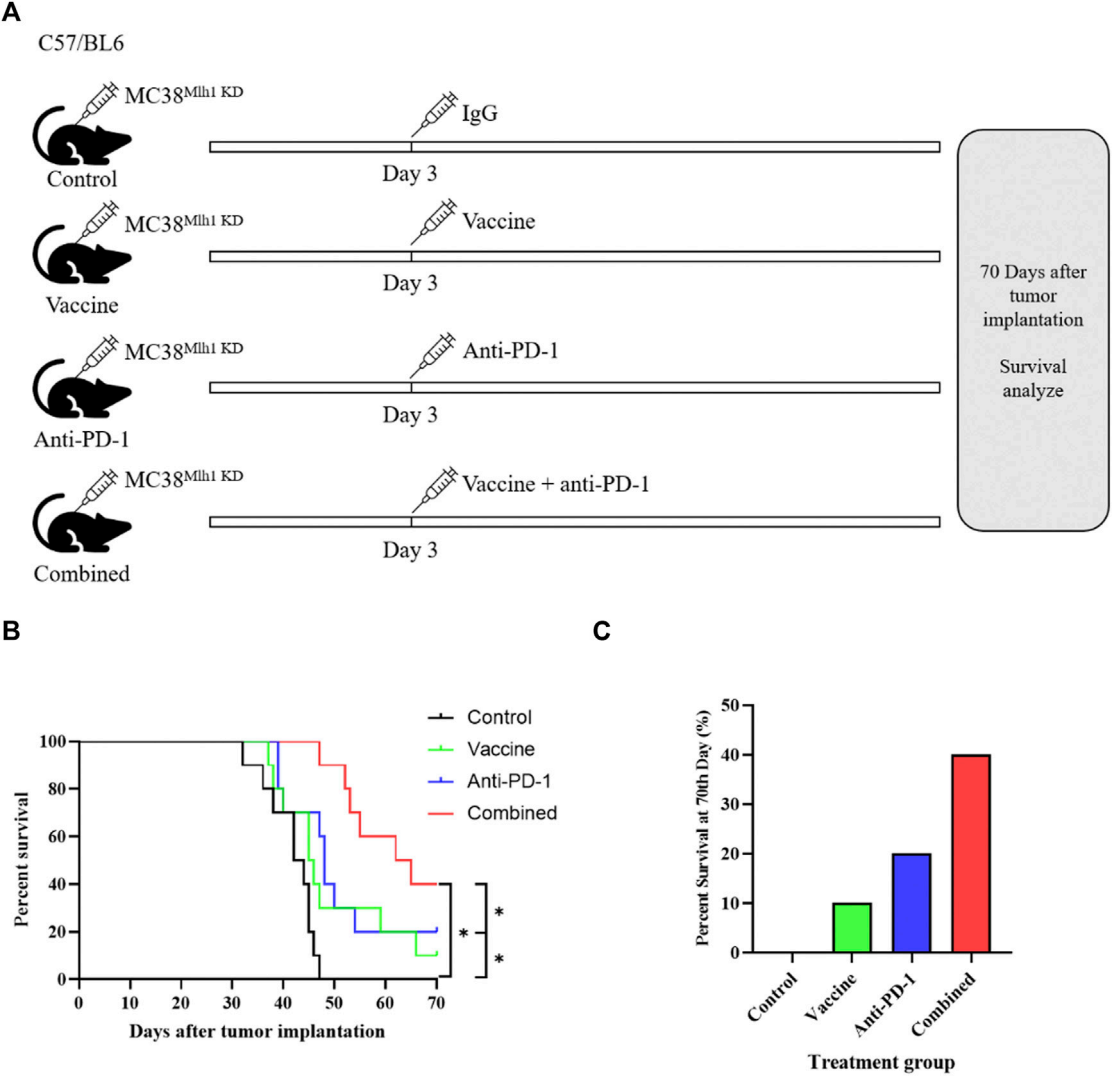
Combination therapy with vaccine and PD-1 blockage improves clinical outcomes in the Lynch syndrome colon cancer mouse model. **(A)** Schema of tumor implantation and treatment (C57/BL6 mice were injected with MC38^Mlh1 KD^ cells in the right lower limb and divided into four groups with ten mice in each group). **(B)** Kaplan-Meier survival curves of mice that were implanted with MC38^Mlh1 KD^ colon cells and were treated with different combinations of IgG, Vaccine and PD-1 antibody. **(C)** The percentages of mice that remained survival at 70th day following tumor implantation and therapy. Statistical significance was measured using Student’s t-test and one-way ANOVA. *, *p* < 0.05.

We first constructed dMMR CRC model cells with stable knockdown of Mlh1 (MC38^Mlh1 KD^), and measured the transfection efficiency through qRT-PCR and western blot. The results exhibited the mRNA and protein expression levels of Mlh1 were significantly decreased in sh-Mlh1 group compared to sh-NC group (Supplementary Figures S1A, B). And then, we injected with 2 × 10^6^ MC38^Mlh1 KD^ cells in the right lower limb of mice to construct an isograft dMMR colon cancer model. In order to verify whether the vaccine effect was specific to the Lynch syndrome model (MC38^Mlh KD^), we treated with vaccine to control group and experimental group. The results exhibited tumor vaccine significantly improve the disease-free survival rate of MC38^Mlh KD^ group mice (Supplementary Figures S2A–C), the tumor volume and weight in MC38^Mlh KD^ group were significantly decreased compared to MC38^Mlh^ wildtype group (Supplementary Figures S2D–F). These results clarified the vaccine effect was specific to the Lynch syndrome model. Subsequently, we explored whether vaccines made by tumor supernatant could increase the anti-tumor activity of PD-1 antibody in Lynch syndrome. Mice were treated with IgG, vaccine, Anti-PD-1 antibodies or vaccine plus Anti-PD-1 antibody on the third day after tumor inoculation in mice vaccinated with MC38^Mlh1 KD^ cells. (Figure 1A). The survival time of mice after treatment demonstrated that with a contrast to anti-PD-1 antibody treatment alone, anti-PD-1 antibody treatment combined with vaccine treatment of mice could improve the overall survival time of tumor mice (Figure 1B). The result also revealed that the combined therapy achieved 70-days survival in more mice than PD-1 antibody or vaccine therapy alone did (Figure 1C).

### Tumor vaccines combined with ICIs can slow the growth rate of tumors in homografted mice

**FIGURE 2:**
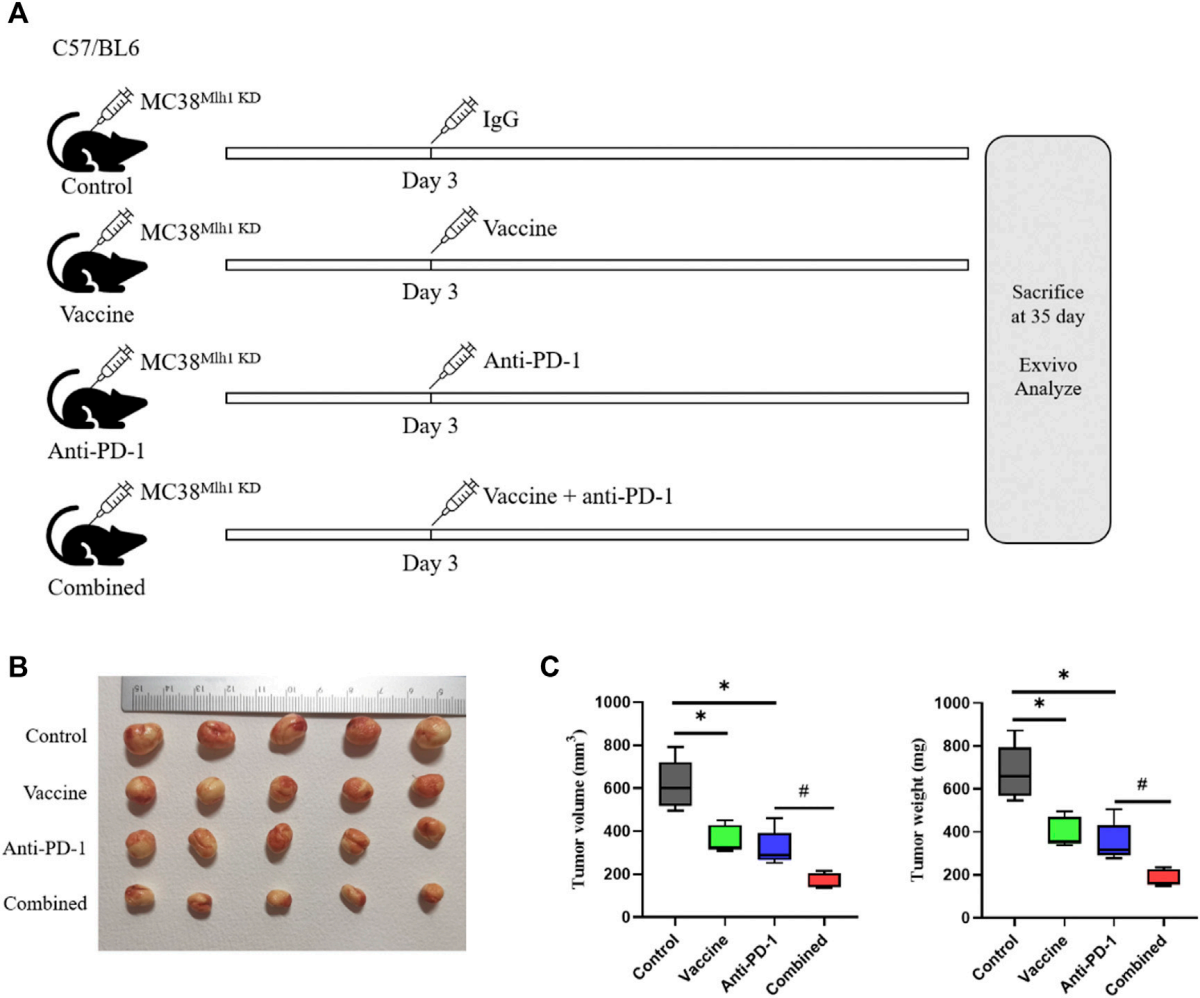
Combination therapy processed better anti-tumor effects than PD-1 blockage and vaccine alone. **(A)** Schema of tumor implantation and treatment (C57/BL6 mice were injected with MC38^Mlh1 KD^ cells in the right lower limb and divided into four groups with five mice in each group). **(B)** Tumors of each group were photographed after being stripped from mice. **(C)** Tumor size and weight were tested after removal from mice. Statistical significance was measured using Student’s t-test and one-way ANOVA. *, *p* < 0.05 vs. Control; ^#^, *p* < 0.05 vs. Anti PD-1.

The above results illustrated that tumor vaccines combined with ICIs could elevate the overall survival time of mice, and we redesigned to treat mice with IgG, vaccine, Anti-PD-1 antibodies or vaccine plus Anti-PD-1 antibody, respectively. Mice were euthanized on day 35, tumor tissues were excised and tumor sizes were measured (Figure 2A). The results demonstrated that the size and weight of tumor tissues in mice were considerably reduced after treatment with vaccine or PD-1 antibody, and the size and weight of tumor tissues could be minimized when treating combined therapy (Figures 2B,C).

### Tumor vaccines can alter the circulatory immune environment in mice

**FIGURE 3:**
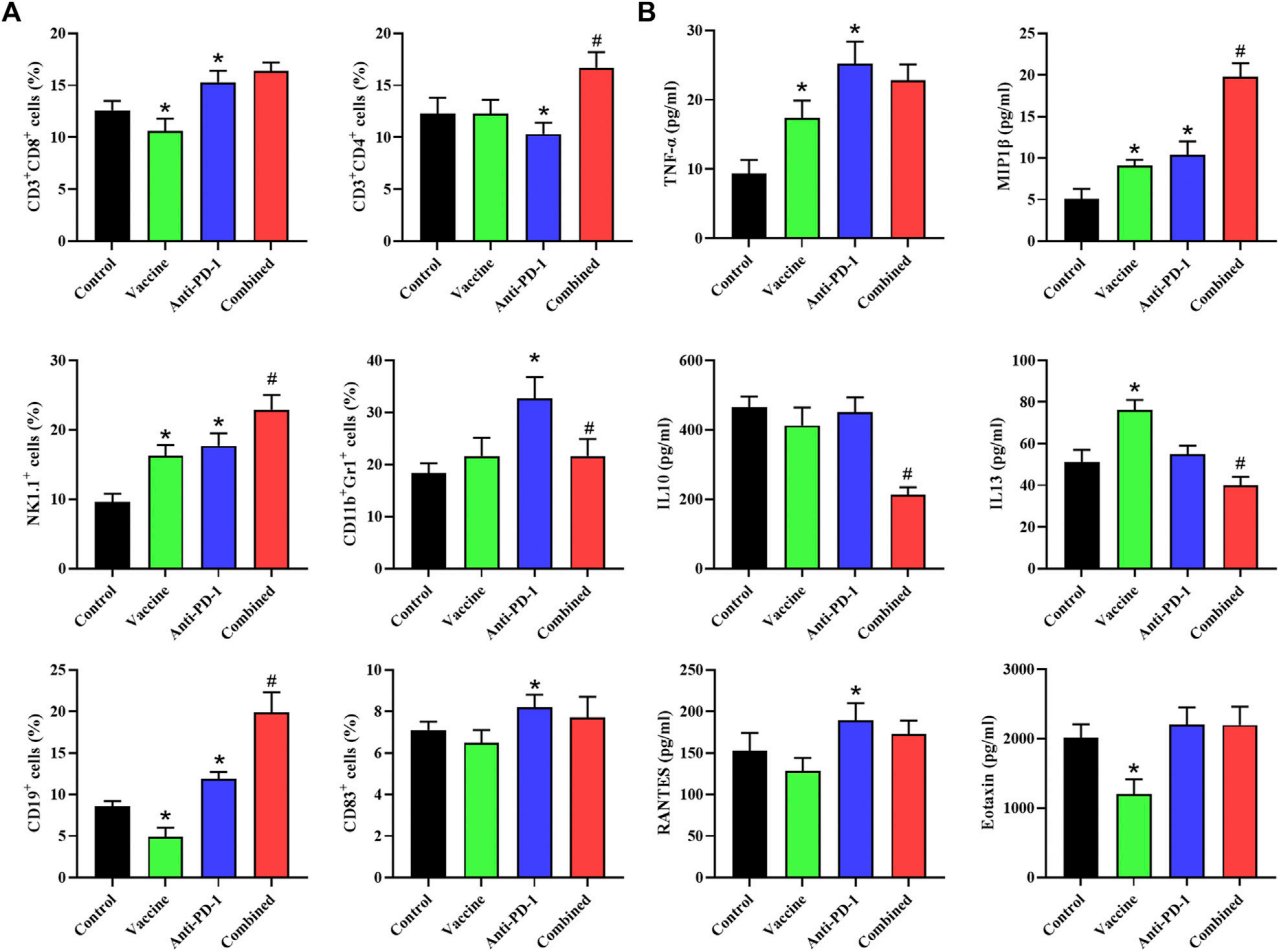
Flow cytometry is used to determine the levels of specific immune cells and chemokines in plasma. **(A)** Phenotyping of peripheral blood leukocytes from mice with different treatment combinations. **(B)** Plasma cytokine levels from mice with different treatment combinations. Statistical significance was measured using Student’s t-test and one-way ANOVA. *, *p* < 0.05 vs. Control; ^#^, *p* < 0.05 vs. Anti PD-1.

Here, we then analyzed the changes in the immune system of mice after treatment. Mouse blood was collected while euthanizing mice, half of which was used for the detection of chemokine content and the rest was used for analysing the content of specific immune cells in the blood using flow cytometry. The results showed that NK cell content was markedly increased, CD19^+^ B cell and CD8^+^ cell contents were evidently decreased after treatment with the vaccine. After PD-1 antibody treatment, the content of CD4^+^ T cells decreased, and the contents of CD8^+^ T, NK, CD11b^+^/Gr1^+^ CD19^+^ as well as CD83^+^ B cells were significantly increased (Figure 3A). The results of combined treatment showed that blood CD4^+^ T cells, NK cells, and CD19^+^ B cells were significantly increased and CD11b^+^/Gr1 cell content was remarkably decreased in mice compared with PD-1 antibody therapy alone (Figure 3A). The results showed that the combination therapy could promote the inflammatory response and improve the effect of PD-1 antibody therapy.

We also detected the contents of chemokines in the serum of mice, and the results suggested that the contents of TNF-α, MIP1β, and IL-13 in the serum were notably increased, and the content of Eotaxin was significantly decreased after treatment with the vaccine. After treatment with PD-1 monoclonal antibody, the levels of TNF-α, MIP1β and RANTES in serum were significantly increased. The content of MIP1β was remarkably increased while the contents of IL-10 and IL-13 were remarkably decreased in the combined treatment group compared with PD-1 monoclonal antibody treatment group (Figure 3B). To sum up, we found that the combination therapy could promote the inflammatory response and reduce the immunosuppression of the circulatory system.

### Cancer vaccines can alter the tumor microenvironment

**FIGURE 4:**
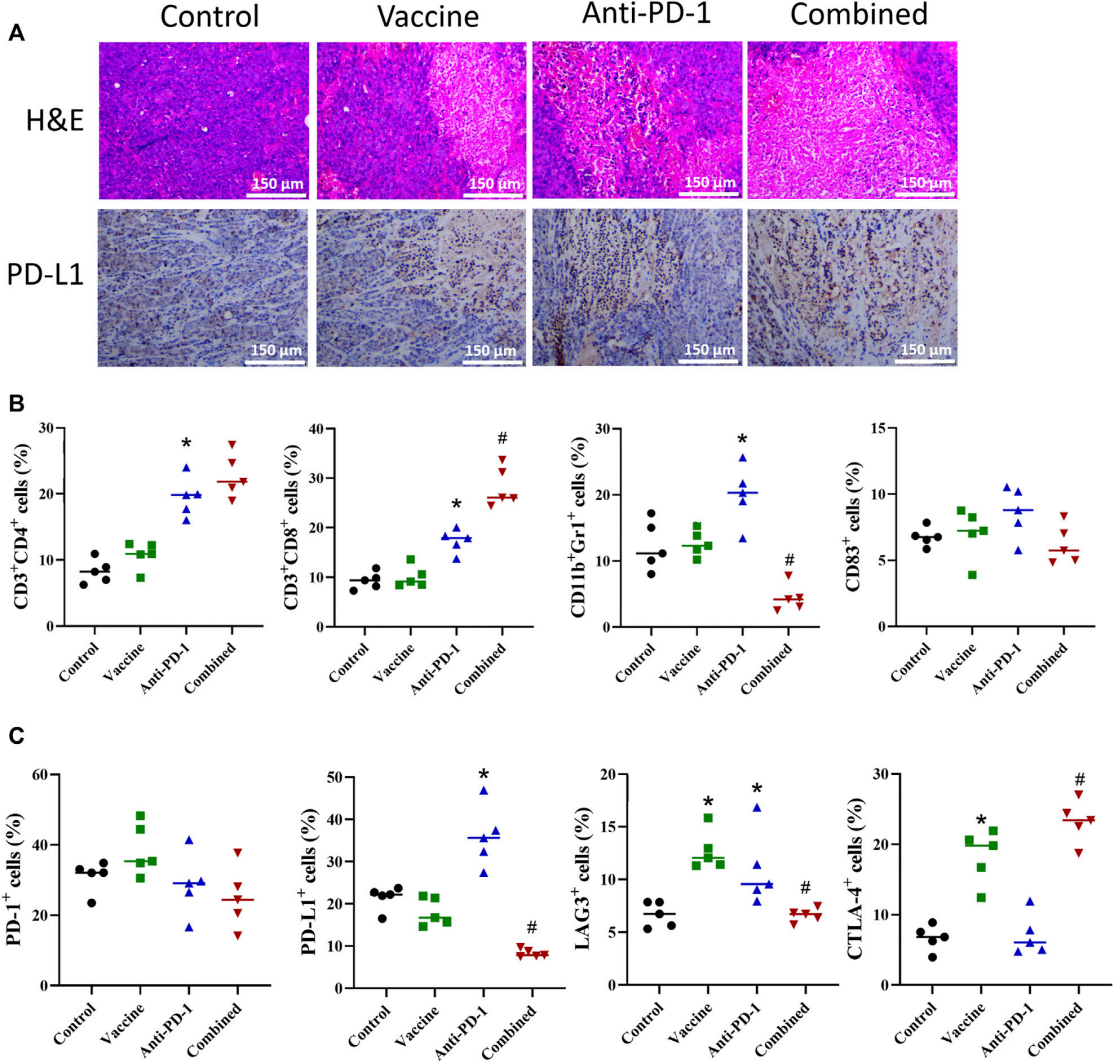
Effect of different combination of treatment on TME. **(A)** Representative H&E staining and IHC from mice with different treatment combinations. **(B–C)** Flow cytometric phenotyping of cells in colon cancer TME. Statistical significance was measured using Student’s t-test and one-way ANOVA. *, *p* < 0.05 vs. Control; ^#^, *p* < 0.05 vs. Anti PD-1.

After exploring the effects of PD-1 antibody, vaccines, and combination therapy on the immune system, we probed into the possible effects of various treatment combinations on the TME. In the study, the tumor tissues of mice were excised, and a part of fresh tissues were taken for IHC and H&E staining. The results of H&E staining demonstrated that the immune cell infiltration in the treatment group was more significant than that in the control group (Figure 4A). The result of PD-L1 IHC staining showed that PD-L1 expression was increased in the vaccine treatment and PD-1 antibody treatment groups, and the combination treatment group exhibited the strongest increasing (Figure 4A). Subsequently, in this study, the cell infiltration in tumor tissues was tested by flow cytometry. The result showed that the increase of various immune cells was not notable after vaccine treatment, while the CD4^+^ T, CD8^+^ T and CD11b^+^/Gr1^+^ cell infiltration was remarkably increased in PD-1 antibody treatment group. Compared with PD-1 antibody treatment group, the CD8^+^ T cell content was significantly increased while CD11b^+^/Gr1^+^ cell infiltration was remarkably decreased in the combined treatment group (Figure 4B). After examining the infiltration of immune-related cells, we also detected the infiltration of cells carrying immune checkpoint-related proteins. The results showed that there was an increase in cells carrying CTL4^+^ and LAG3^+^ proteins after treatment with vaccine, and there was a significant increase in cells carrying PD-L1 and LAG3 proteins after PD-1 antibody treatment. The results showed that both vaccine treatment and PD-1 antibody treatment caused stress synthesis of immunosuppressive proteins in cancer cells (Figure 4C). What’s more, the results of combination therapy showed that compared with PD-1 antibody therapy alone, the expression of PD-L1 and LAG3 in tumor tissues were markedly decreased, and the expression of CTLA-4 was significantly increased. These data showed that the combination therapy exhibited a better inhibitory effect on immune escape than PD-1 monoclonal antibody therapy alone (Figure 4C).

### Tumor vaccine combined with ICIs inhibited the proliferation of tumor tissue and angiogenesis

**FIGURE 5:**
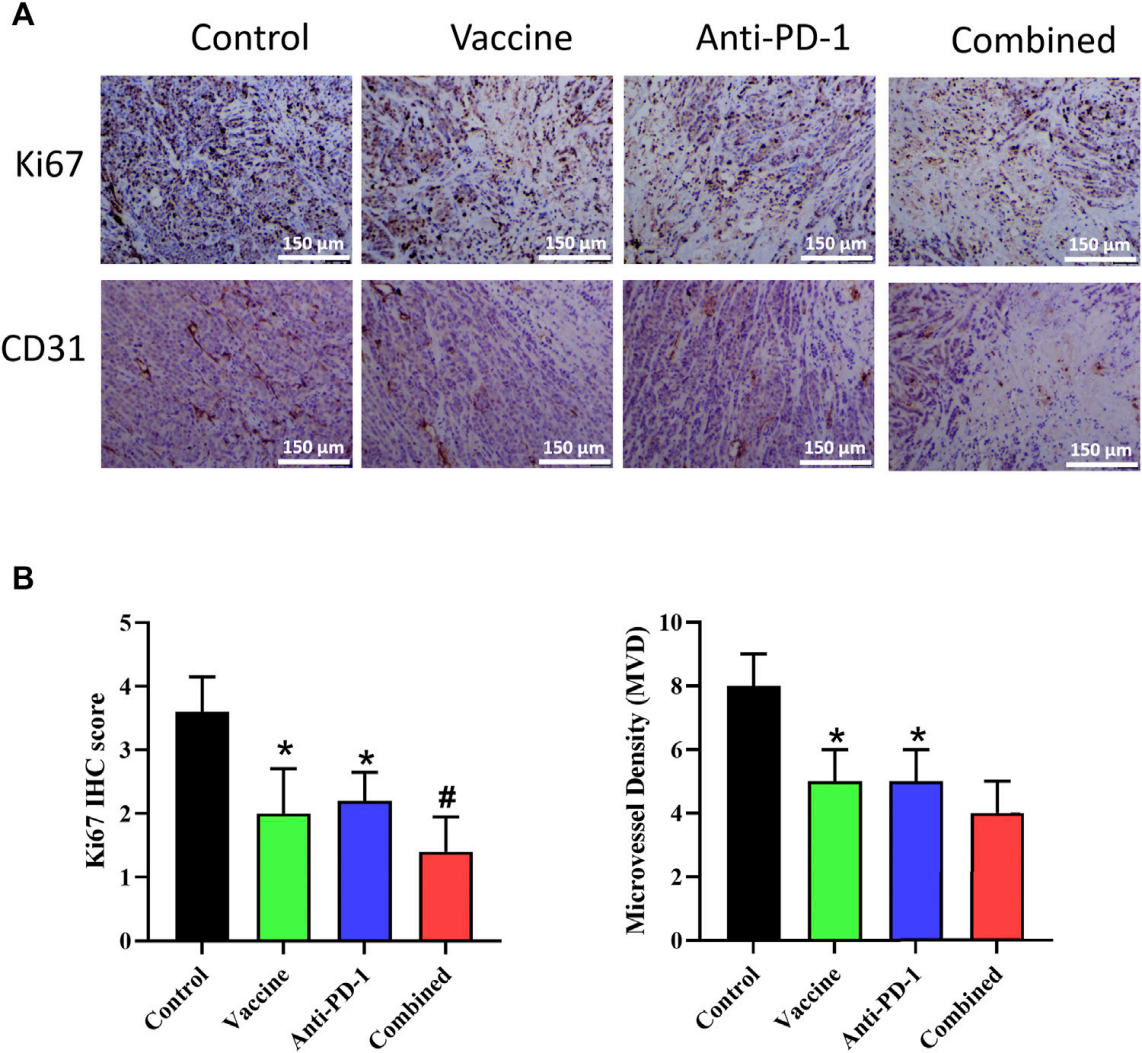
Effect of vaccine, PD-1 blockage and combination therapy on cellular proliferation, and vessel density in tumors. **(A,B)** Ki67^+^ cells and microvessel density are quantified in mice treated with different therapy. Statistical significance was measured using Student’s t-test and one-way ANOVA. *, *p* < 0.05 vs. Control; #, *p* < 0.05 vs. Anti PD-1.

To further explore the effect of vaccine, PD-1 antibody and combination therapy on the treatment of Lynch syndrome, we applied IHC to detect the proliferation and angiogenesis of tumor tissues in mice. The result of Ki67 expression demonstrated that vaccine, PD-1 antibody, and combination therapy treatment could reduce the level of tumor cell proliferation in Lynch syndrome, and combination therapy acted the best effect (Figures 5A,B). The results of CD31 staining illustrated that vaccine, PD-1 antibody and combination therapy treatment reduced angiogenesis in Lynch syndrome mouse model, and combination therapy exhibited the best effect (Figures 5A,B). The above results suggested that vaccine and PD-1 antibody treatment could inhibit the proliferation level of tumor cells and reduce angiogenesis in tumor tissues, combination therapy treatment exerted the best inhibitory effect.

## Discussion

Cancer therapy for patients with Lynch syndrome has been a tricky point, and the advent of immunotherapy, especially ICIs, has enormously improved the survival of patients with Lynch syndrome (Lee and Le, 2016; Winer et al., 2019). Because of the strong immunogenicity of tumor tissues in patients with Lynch syndrome, patients have a great response to ICIs (Cerretelli et al., 2020). At present, in addition to the use of ICIs for treating cancer patients with Lynch syndrome, cancer vaccine is also an effective treatment method. At present, many studies have attempted to use tumor vaccines to prevent the incidence of tumors in patients with Lynch syndrome. For example, researchers have discovered that injection of immunogenic frameshiftpeptides (FSP) into patients with Lynch syndrome can prevent the incidence of Lynch syndrome-related cancers (von Knebel Doeberitz and Kloor, 2013; Majumder et al., 2018). In addition, tumor antigen peptide vaccines prepared by using tumor-specific antigens can directly kill tumor tissue or improve the therapeutic effect of other immunotherapies by stimulating the patient’s immune system and recruiting killer immune cells (Soares et al., 2015). At present, a large number of clinical trials related to cancer vaccines have been carried out, and encouraging results have been obtained in melanoma (Carreno et al., 2015; Li et al., 2017). In this study, we firstly clarified that tumor vaccine effect was specific to the Lynch syndrome model (MC38^Mlh KD^). Besides, we prepared MC38^Mlh1KD^ Lynch syndrome model cells into cell lysate vaccine and treated homograft mice with different methods, and found that tumor vaccine combined with PD-1 blocking therapy could increase the survival rate of mice and prolong the overall survival time of tumor-bearing mice. Subsequent experiments also revealed that the combined treatment could reduce the growth rate of tumor tissues in mice. The current study found that combining tumor vaccines with immunotherapy can hinder tumor growth by improving the therapeutic effect of traditional immunotherapy. For example, the team of Duraiswamy et al. (2013) *.* combined tumor vaccines with PD-1/CTLA-4 blockage to successfully improve the activity of effector T cells in mouse tumor cells, weaken the inhibitory effect of T-reg and strengthen the therapeutic effect of PD-1/CTLA-4 blockage. Herein, we found that tumor vaccines prepared from Lynch syndrome cell lysates could assist the therapeutic effect of ICIs and markedly improve the survival time of tumor-bearing mice.

The TME is a collection of a series of cells in tumor tissues such as tumor cells, mechanistic cells, and immune cells, which can affect the growth of tumor tissues in a variety of ways, such as lower oxygen content can inhibit the immune response and promote the division of tumor tissues, in addition, lower pH can also curb the activity of immune cells, attenuate the effect of chemotherapeutic drugs, and facilitate immune escape of tumors (Vanichapol et al., 2018; Lei et al., 2020). Since the TME has a significant and auxiliary part in the growth of tumor tissue, its targeting role in cancer therapy cannot be disregarded. There are many current treatments to target the TME, such as the study team using the combination therapy of Apatinib to promote the immune response of the immune system by improving blood supply to tumor tissues and reducing the hypoxic condition of tumor tissues (Zhao et al., 2019). In addition, there are pH-sensitive nanomedicines designed using the tumor pH microenvironment to inhibit tumor development by inducing apoptosis in tumor tissue (Wijesinghe et al., 2013). Recent studies have found that tumor vaccines are an effective means to improve the TME and avoid immune escape from tumor tissues, for example, Xu et al. (2020) *.*s team regulated the TME through Listeria-based hepatocellular carcinoma vaccine and induced macrophage differentiation to M1 phenotype to promote immune system killing of tumor tissues. In this study, we examined the immune infiltration in the circulatory system and TME of homograft mice treated with tumor vaccine using flow cytometry and ELISA, and found that killer immune cells such as CD8, CD4, and NK were significantly increased after treatment, while immunosuppressive cells MDSC and immunosuppressive cells with PD-1 and LAG3 antigens were increased after PD-1treatment and significantly decreased after vaccine combination therapy. This suggests that tumor vaccines can reverse the drug resistance response of tumor tissues to PD-1 treatment and inhibit the resistance of tumor tissues to PD-1 blocking therapy. In addition, we detected the contents of immune factors and cytokines in the circulatory system of mice after vaccine treatment, and the results demonstrated that the contents of immunosuppressive cytokines were notably reduced in the combined treatment group, and the expression levels of inflammatory cytokines such as TNF-α and MIP-1β were remarkably elevated in the combined treatment group. In summary, this study found that cancer vaccines can increase the anti-tumor immune cell content in tumor tissues by improving the TME and regulating immune infiltration, which can not only inhibit the development of cancer itself, but also synergize ICIs immunotherapy.

Angiogenesis of the TME and proliferation of tumor tissue are the main causes of rapid tumor development (Zhao et al., 2019). Therefore, this study also examined the effects of immunotherapy as well as vaccine therapy on tumor cell proliferation and tumor tissue angiogenesis, and the results showed that vaccine therapy could enhance the inhibitory effect of immunotherapy on tumor cell proliferation and improve the inhibitory effect of immunotherapy on angiogenesis. In this study, we found that the combination therapy could greatly inhibit the proliferation of tumor cells and the angiogenesis of tumor tissues, thus inhibiting the growth of tumor tissues.

In summary, this paper found that tumor vaccines had a therapeutic effect on colon cancer induced by Lynch syndrome, which can also assist ICIs therapy to improve the therapeutic effect of ICIs. At the same time, tumor vaccines could regulate the TME, increase immune infiltration in tumor tissues, reduce the proportion of immunosuppressive cells, inhibit tumor growth by inhibiting tumor cell proliferation and angiogenesis in tumor tissues, and exert a therapeutic role in colon cancer caused by Lynch syndrome. Tumor vaccine was confirmed in our study to have a great therapeutic effect on colon cancer caused by Lynch syndrome. The therapeutic effect of ICIs treatment was improved by tumor vaccines and investigated by animal experiments. This study provides a reliable theoretical reference for the treatment of related cancers caused by Lynch syndrome. Although this study is enough to prove the role of cancer vaccines in treatment, there are still shortcomings. In this study, we applied the Lynch syndrome model with MC38^Mlh1 KD^ cells injected into the limb of mice, which lacked the content of the native colon cancer environment. We would try to construct modeling grafted colon cancer with a proper microenvironment in the follow-up study. Due to conditions and other reasons, this paper does not well simulate the actual incidence of Lynch syndrome, and does not further explore the molecular mechanism by which the vaccine works. The team intends to further construct an *in vivo* immune reconstitution model and further study the effect of tumor vaccines and the molecular mechanism using techniques such as gene editing on a model closer to Lynch syndrome.

## Data Availability

The original contributions presented in the study are included in the article/Supplementary Material, further inquiries can be directed to the corresponding authors.
