# Supplementary figures and images for: PD-1 blockage combined with vaccine therapy can facilitate immune infiltration in tumor microenvironment of Lynch syndrome colon cancer
Source: Front Genet. 2022 Sep 8;13:877833. doi: 10.3389/fgene.2022.877833 (PMC9493022; doi:10.3389/fgene.2022.877833)

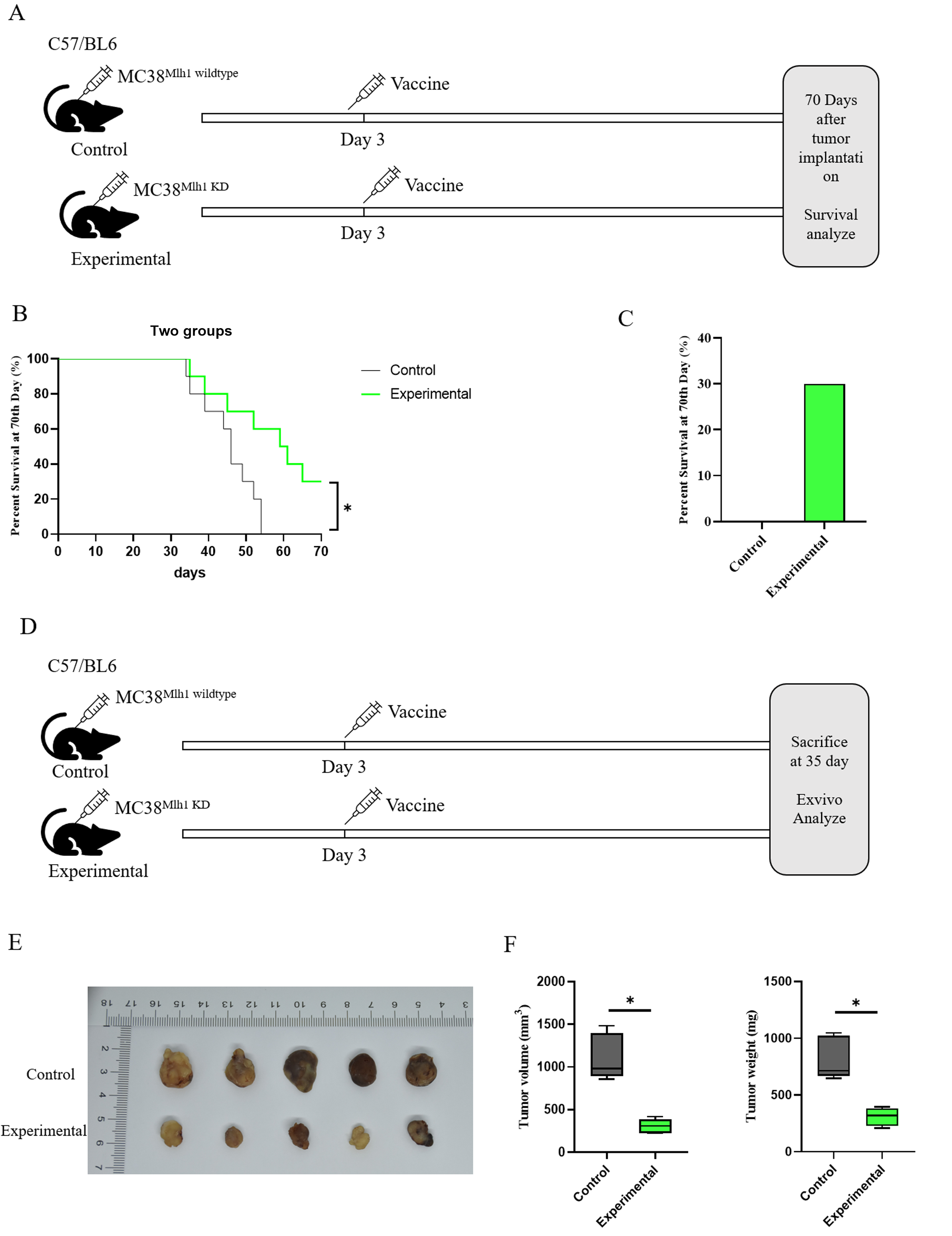

Supplement: Supplementary file 1 [file Image2.tif]

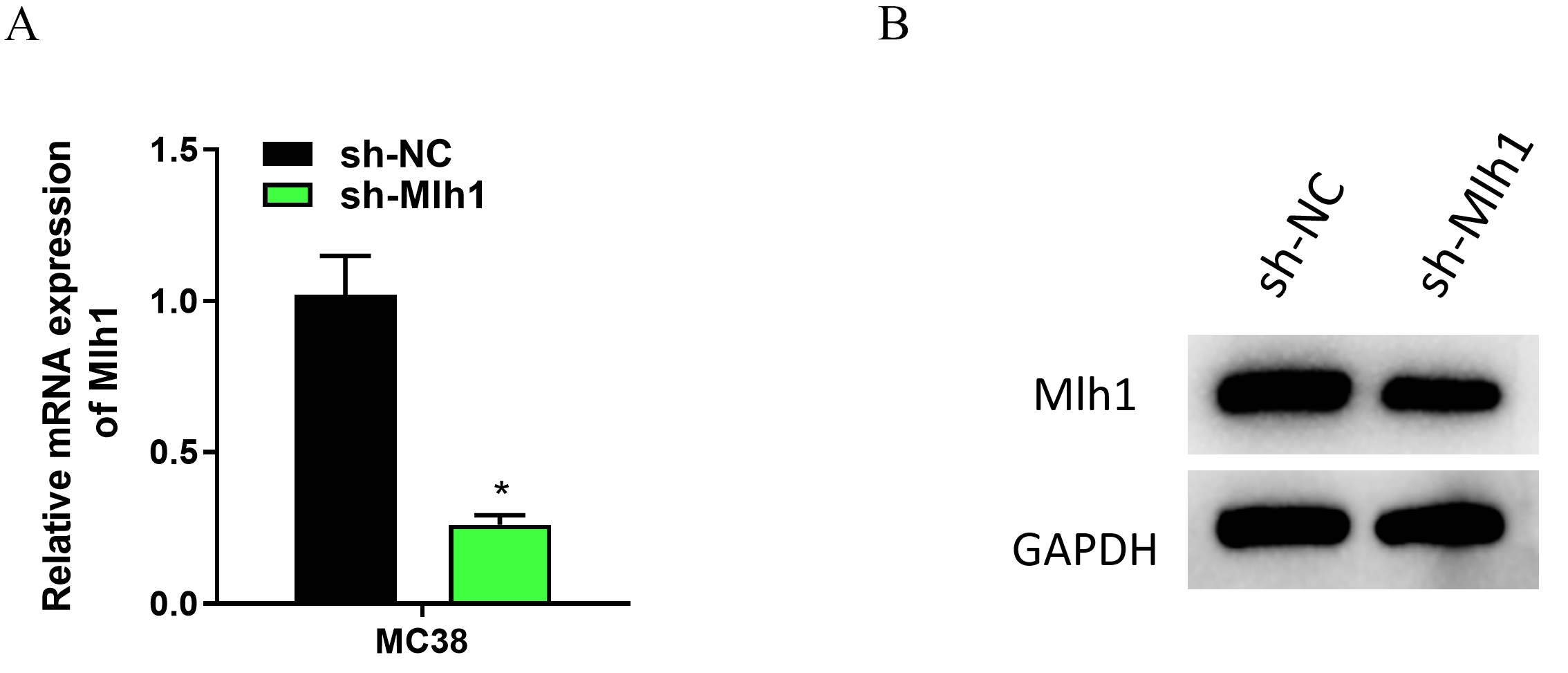

Supplement: Supplementary file 2 [file Image1.tif]
